# The First Genome of the Cold-Water Octocoral, the Pink Sea Fan, *Eunicella verrucosa*

**DOI:** 10.1093/gbe/evad083

**Published:** 2023-05-20

**Authors:** Kirsty L Macleod, Josephine R Paris, Tom L Jenkins, Jamie R Stevens

**Affiliations:** Department of Biosciences, Faculty of Health and Life Sciences, University of Exeter, Exeter, United Kingdom; Department of Biosciences, Faculty of Health and Life Sciences, University of Exeter, Exeter, United Kingdom; Department of Biosciences, Faculty of Health and Life Sciences, University of Exeter, Exeter, United Kingdom; Department of Biosciences, Faculty of Health and Life Sciences, University of Exeter, Exeter, United Kingdom

**Keywords:** octocoral, cold-water coral, genome, *Eunicella verrucosa*, long-read sequencing

## Abstract

Cold-water corals form an important part of temperate benthic ecosystems by increasing three-dimensionality and providing an important ecological substrate for other benthic fauna. However, the fragile three-dimensional structure and life-history characteristics of cold-water corals can leave populations vulnerable to anthropogenic disturbance. Meanwhile, the ability of temperate octocorals, particularly shallow-water species, to respond to adjustments in their environment linked to climate change has not been studied. This study reports the first genome assembly of the pink sea fan (*Eunicella verrucosa*), a temperate shallow-water octocoral species. We produced an assembly of 467 Mb, comprising 4,277 contigs and an N50 of 250,417 bp. In total, 213 Mb (45.96% of the genome) comprised repetitive sequences. Annotation of the genome using RNA-seq data derived from polyp tissue and gorgonin skeleton resulted in 36,099 protein-coding genes after 90% similarity clustering, capturing 92.2% of the complete Benchmarking Universal Single-Copy Orthologs (BUSCO) ortholog benchmark genes. Functional annotation of the proteome using orthology inference identified 25,419 annotated genes. This genome adds to the very few genomic resources currently available in the octocoral community and represents a key step in allowing scientists to investigate the genomic and transcriptomic responses of octocorals to climate change.

**Significance Statement**

In contrast to many tropical coral species, very little is known about the ability of cold-water corals to adapt to changes in their environment associated with climate change, particularly elevated seawater temperature. Yet, cold-water corals comprise more than half of the coral species that exist today and perform key ecological roles throughout the habitats in which they are found, most importantly by increasing the three-dimensional structure of temperate ecosystems where reef-building corals are absent. The lack of genomic resources for cold-water corals has limited investigations into the potential for these species to adapt to environmental perturbations on a genomic level; the genome of the pink sea fan, *Eunicella verrucosa*, will be a vital tool for the cold-water coral community in pursuing such questions.

## Introduction

The pink sea fan, *Eunicella verrucosa*, is a temperate octocoral within the soft coral order Malacalcyonacea (formerly Alcyonacea; see McFadden et al. 2022) and a member of the Gorgoniidae family. This species is distributed across the northeast Atlantic from western Ireland to (reportedly) the coast of Mauritania in West Africa (Hayward and Ryland 2017) and as far east as the Aegean Sea (Chimienti et al. 2020). The species is mostly found in dense “forest-like” aggregations (Chimienti et al. 2020; Jenkins and Stevens 2022), while at its range-edge, for example, Pembrokeshire, southwest Wales (Holland et al. 2017), it exhibits a patchy distribution. Its depth ranges from 3 to 50 m within the northeast Atlantic (Readman and Hiscock 2017) and down to 200 m in the Mediterranean Sea (Sartoretto and Francour 2012; Chimienti et al. 2019).

Gorgonians often act as key ecological substrates for many epifauna, increasing the structural complexity of benthic ecosystems (Wood 2013; Pikesley et al. 2016). For *E. verrucosa*, this species' slow growth (Coz et al. 2012), longevity, and physical three-dimensional structure can render local populations vulnerable to ecological pressures, including physical disturbance (Readman and Hiscock 2017) and disease (Hall-Spencer et al. 2007). Given the current distribution of *E. verrucosa*, and observations of the relationship between thermal regime and distribution in other octocorals (Ferrier-Pages et al. 2009; Haguenauer et al. 2013; Arizmendi-Mejía et al. 2015; Crisci et al. 2017; Oualid et al. 2023), seawater temperature may be a key pressure underpinning local population persistence. Despite this, a dedicated study exploring this has not been conducted (although see Jenkins and Stevens 2022), whilst genomic and transcriptomic analyses have been limited due to the lack of a genome for the species.

Across most of its range, *E. verrucosa* is protected under the EU Habitats Directive Annex 1 and under Ecologically or Biologically Significant Marine Areas (EBSAs) throughout the Mediterranean Sea. In the United Kingdom, it is a “protected feature” used for the designation of Marine Protected Areas (MPAs) and previous research into genetic connectivity across southwest Britain (Holland et al. 2013, 2017) has been used to assess whether MPAs represent an “ecologically coherent” network (Jenkins and Stevens 2018).

Ecological research questions are increasingly focused on the adaptive potential of marine taxa to environmental change and how subsequent conservation measures, such as MPA designations, can be more resilient to future anthropogenic and ecological pressures (Donelson et al. 2019; Hoppit et al. 2022). Very few genomic resources for octocorals are currently available, hindering such investigations into their adaptive capacity and the implications this may have for effective conservation and mitigation practices. This report presents the first annotated genome of a pink sea fan that will augment the limited genomic resources available in octocoral research, allowing scientists to investigate hypotheses concerning the species' potential responses to environmental change.

## Results and Discussion

### Assembly

**Fig. 1:**
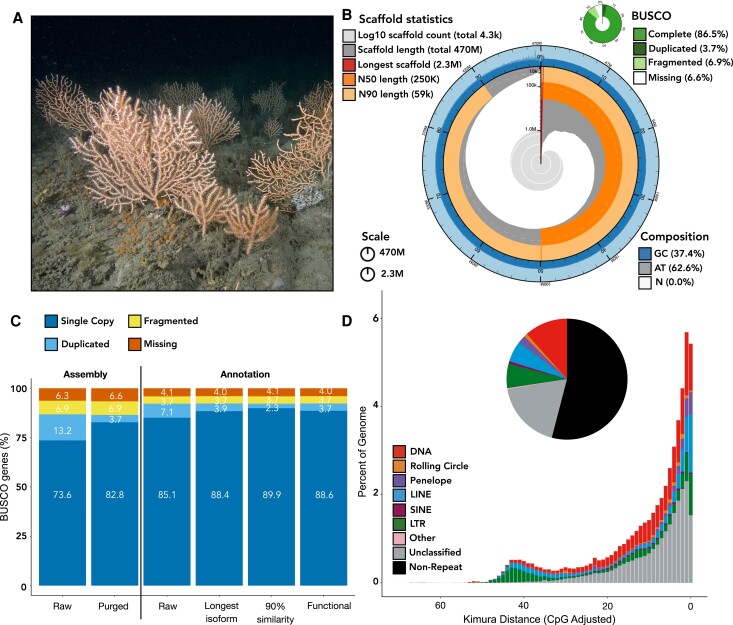
(*A*) *Eunicella verrucosa* colonies from Plymouth, southwest England, showing the three-dimensionality of populations and variation in colony shape and size; photo credit: Dr Paul Naylor. (*B*) Snail plot summarizing the final genome assembly summary statistics and the composition of BUSCO genes. (*C*) BUSCO statistics for the genome assembly and annotation sets. The purged assembly (removal of 6,766 haplotigs) comprised fewer duplicated BUSCO genes than the raw genome assembly. Filtering the raw annotation (longest isoform and 90% similarity) increased BUSCO completeness with the final annotation gene set containing 89.9% single-copy genes and fewer duplicated BUSCO genes (2.3%). (*D*) Summary of the transposable element families detected using Earl Grey comprising 45.96% of the genome. The proportion of nonrepeat sequences (54.04%) represent the remaining genomic DNA sequences and are comparable with other cold-water corals (see supplementary table S2, Supplementary Material online, for full summary statistics).

We generated 29.96 GB (∼46.8-fold coverage) of PacBio circular consensus reads (>1 kb in length), producing an initial genome assembly of 467 Mb comprising 11,043 contigs with an N50 of 183,250 bp (“Raw” assembly—fig. 1*C*). After identification and removal of 6,766 haplotigs (supplementary fig. S1, Supplementary Material online), we produced a final assembly with 4,277 contigs, with an improved N50 of 250,417 bp (fig. 1*B*; “Purged” assembly—fig. 1*C*). Preliminary Benchmarking Universal Single-Copy Orthologs (BUSCO) assessment using the metazoan conserved orthologs (*n* = 954) showed a completeness score of 86.5% (82.8% single-copy, 3.7% duplicated, 6.9% fragmented, and 6.6% missing) (fig. 1*B*and 1*C*). In comparison with the very few available octocoral genomes, the number of contigs suggests a more contiguous assembly than that of *Paramuricea clavata* and fewer missing BUSCO genes than the assemblies of *P. clavata* and *Trachythela* sp. (table 1).

**Table 1:** Assembly Statistics for Published Genomes of Other Cnidarians

Species	Total size (Mb)	No. of contigs	Contig N50(bp)	BUSCO analysis (%)					Repeat elements (%)	No. of genes
				*C*	*S*	*D*	*F*	*M*		
*Octocoral*										
*E. verrucosa* *(this study)*	467	11,043	183,250	86.5	82.8	3.7	6.9	6.6	45.96	36,099
*P. clavata (Ledoux et al. 2020)*	607	64,145	19,152	75.8	73.4	2.4	9.4	14.8	49	62,652
*D. gigantean (Jeon et al. 2019)*	276	—	1,445,523	93.9	87.3	6.6	2.4	3.5	12	28,879
*Trachythela* sp. *(Zhou et al. 2020)*	578.26	396	3,563,727	90.7	88.4	2.3	2.0	7.3	58.9	35,305
*Stony coral*										
*S. pistillata (Voolstra et al. 2017)*	434	32,144	24,388	^a^94.76 (*gene database: 248 CEGs*)					28.8	25,769

For *P. clavata* and *D. gigantean,* BUSCO completeness was assessed using the 978 metazoan gene database; the genome of *Trachythela* sp. was assessed using the 954 metazoan gene database. BUSCO gene abbreviations: *C*, complete; *S*, single-copy; *D*, duplicated; *F*, fragmented, and *M*, missing.

a Gene completeness for the *S. pistillata* genome was assessed with 248 Core Eukaryotic Genes (CEGs).

We identified 213 Mb (45.96%) of repetitive sequence in the genome assembly, of which 18% comprised unclassified repeats and the remaining 27% were categorized into repeat families, the highest being DNA repeats (∼12%) (fig. 1*D* and supplementary table S2, Supplementary Material online). This is comparable with the genomes of *P. clavata* (Ledoux et al. 2020) and *Trachythela* sp. (Zhou et al. 2020), which had 49% and 58.9% of the genome composed of repetitive elements, respectively (table 1). When compared with the genome of *Dendronephthya gigantean* (12% repetitive elements), a much greater number of repetitive elements were identified in *E. verrucosa*, but *D. gigantean* has a considerably smaller assembly size (table 1).

### Annotation

We performed gene prediction using paired-end RNA-seq data. The initial annotation (“Raw” annotation—fig. 1*C*) was produced using the initial gene predictions, recovering 41,933 genes. BUSCO analysis showed a high proteome contiguity and completeness: 92.2% complete BUSCO genes (85.1% single-copy, 7.1% duplicated, 3.7% fragmented, and 4.1% missing) but a high number of duplicated genes (fig. 1*C* and supplementary table S2, Supplementary Material online), indicating redundancy in this initial gene set. Filtering this initial proteome for the longest isoform reduced the BUSCO gene duplication from 7.1% to 3.9% (“Longest isoform” annotation—fig. 1*C* and supplementary table S1, Supplementary Material online). Despite this, the gene annotation was still higher than expected (40,003 protein-coding genes), especially given the number of genes annotated in other octocorals (table 1). Annotations were therefore filtered for 90% clustering similarity, removing 3,904 genes and resulting in an annotation containing 36,099 genes (“90% similarity” annotation—fig. 1*C*). This final annotation contained 92.2% complete BUSCOs and lowered the duplication rate further from 3.9% to 2.3% (fig. 1*C*). Overall, this indicates that the *E. verrucosa* proteome has the second highest BUSCO gene completeness compared with other available octocoral proteomes (table 1). Functional annotation of the final annotation using eggNOG-mapper and InterProScan identified 25,419 functionally annotated genes, containing 92.3% complete BUSCO genes (88.6% single-copy, 3.7% duplicated, 3.7% fragmented, and 4.0% missing). All versions of these gene annotations are available on GitHub (https://github.com/klmacleod/pinkseafan-genome).

### Comparative Proteome Analysis

Using our annotation data sets, we performed a comparative analysis using other available octocoral proteomes. Filtering of *D. gigantean* and *Stylophora pistillata* annotation sequences at 90% sequence similarity using CD-HIT produced a reference Blast database of 18,649 and 22,553 genes, respectively. BlastP of the *E. verrucosa* proteome against both reference proteomes, using an *e*-value cut-off 0.00001 and then filtering for a 95% overlap hit ratio, gave 30,308 hits against *D. gigantean* and 25,876 hits for *S. pistillata*. Protein comparison of shared functional genes, annotated via eggNOG-mapper and InterProScan, indicated that 23,827 (93.7%) genes were present in all three species, with a greater proportion of genes (998 genes; 3.9%) shared between *E. verrucosa* and the closely related octocoral *D. gigantean*, whereas only 251 genes (1%) were common to both *E. verrucosa* and *S. pistillata* (supplementary fig. S2, Supplementary Material online). A total of 343 genes (1.3%) were unique to the proteome of *E. verrucosa*.

This first annotated genome of *E. verrucosa* represents a key tool in supporting future investigation into any potential genomic and transcriptomic mechanisms underpinning octocoral responses to environmental change and may also aid comparative analysis between octocorals and the more widely studied tropical, stony corals.

## Materials and Methods

### Sample Collection

A detailed description of the sample collection can be found in the Supplementary material. Six colonies were collected via SCUBA at 8.5–12.7 meters depth in Plymouth Sound, England (lat. 50.33, long. −4.14) (L/2019/00143), representing the northern region of the species' global distribution. Colonies were transported in chilled seawater to a 350-l artificial seawater tank at the Aquatic Resources Centre, University of Exeter. Colonies were left to acclimate for 19 months at 14.3 °C (+/−0.5 °C) degrees prior to DNA extraction.

### Genomic DNA Extraction, Library Preparation, and Sequencing

Extracting sufficient quality and quantity of DNA and RNA from octocoral polyps is notoriously challenging. Whilst the underlying reasons for this is not well-understood, we have dedicated significant efforts to optimize extraction (detailed protocols are available in the Supplementary material). Briefly, genomic DNA was extracted from polyp tissue using a salting-out protocol (Jenkins et al. 2019) optimized for the semi-rigid, gorgonian protein tissue of *E. verrucosa*. DNA extraction integrity was assessed on a 1% agarose gel, purity using a NanoDrop 1000 spectrophotometer, and concentration using the Invitrogen dsDNA HS Assay kit and a Qubit 4 Fluorometer. DNA extractions were cleaned using the Qiagen DNeasy PowerClean Pro Cleanup Kit according to the manufacturer protocol until DNA precipitation, which was performed using isopropanol and then elution of genomic DNA via the salting-out protocol.

The quality of genomic DNA was checked on a pulsed field gel (Bio-Rad Chef-DR II). SMRTbell libraries were prepared using a SMRTbell Express Template Prep Kit 2.0 (Pacific Biosciences) including a size selection of 15 Kb or greater using a BluePippin (BPLUS10, Sage Science). The library was diffusion loaded at 5 pM on the PacBio Sequel I using SMRTcell 1Mv3. Data were sequenced across three SMRT cells (expected output range of 120–180 Gb). Sequencing was carried out by the University of Exeter Sequencing Service.

### Genome Assembly and Quality Assessment

An estimate for the expected size of the *E. verrucosa* genome size was not available, and we did not generate short-read data to estimate the genome size using *kmer* profiling. We therefore used the *C*-value of the only soft coral, *Sarcophyton* sp. (640 Mbp; Adachi et al. 2017) as a guide for the expected genome assembly span.

The genome was assembled using the assembly algorithm in Flye v2.8.3 (Kolmogorov et al. 2019) with default settings for PacBio long reads. Based on initial assessment of assembly contiguity and BUSCO completeness, reads shorter than 1,000 bp were removed from the initial assembly. Contamination of contigs was assessed using Blobtools v1.1.1 (Laetsch et al. 2020), but no evidence of contamination was found (supplementary figs. S3, and S4, Supplementary Material online). Duplicated haplotigs were assessed and removed using Purge_dups v1.2.6 (Guan et al. 2020). To evaluate the assembly, presence and completeness of orthologs were assessed using BUSCO v5.1.3 (Simão et al. 2015) using the metazoan database containing 954 genes.

### Repetitive Sequence Identification

We used Earl Grey v1.3 (Baril et al. 2022), an automated transposable element (TE) annotation pipeline, which uses RepeatModeler v2.0.2 (Flynn et al. 2020) and RepeatMasker v4.1.2 (Tarailo-Graovac and Chen 2009) to identify repeats. Earl Grey combines pre-existing library-based and de novo TE annotation tools, but performs TE consensus and annotation refinements leading to fewer erroneous estimates of TE count and longer consensus sequences (Baril et al. 2022). The complete Dfam library (version 3.6; Dfam-p1.h5) was used for RepeatMasker (Storer et al. 2021).

### RNA-Seq Extraction, Library Preparation, and Sequencing

RNA-seq data were collected from *E. verrucosa* colonies which had undergone an ex situ thermal experiment. Briefly, 10 cm fragments were individually exposed to thermal regimes representing the minima and maxima across the species' range. After 24 h, whole fragments were flash frozen in liquid nitrogen and stored at −80 °C. In total, 24 fragments were sampled. RNA extraction was conducted using QIAzol Lysis Reagent (QIAGEN) and 1-bromo-3-chloropropane (BCP), and RNA-seq libraries were prepared from total RNA using the NEB Next® Ultra™ RNA Library Prep Kit (see Supplementary Materials). Quantified libraries were pooled and paired-end sequenced on a NovaSeq 6000 S4 flow cell (Illumina, Inc).

### Genome Annotation

Prior to annotation, the genome assembly was softmasked for repeats (identified with Earl Grey) using BEDTools v2.27.1 (Quinlan and Hall 2010). RNA-seq reads were mapped to the softmasked assembly using the splice-aware aligner STAR v2.7.3 (Dobin et al. 2013). Annotation was carried out using the BRAKER2 v2.1.2 automated annotation pipeline (Brůna et al. 2021), and ab initio gene prediction was performed from spliced aligned RNA-seq reads using Augustus v3.5.0 (Stanke et al. 2006). Two filtering methods were used to assess the quality of the genome annotation. Firstly, the longest isoform and corresponding protein sequences were extracted using *agat_sp_keep_longest_isoform.pl* and *agat_sp_extract_sequences.pl* from AGAT v.1.0 (Dainat 2022). Secondly, to investigate possible gene redundancy, annotation sequences were clustered by 90% similarity using CD-HIT v4.8.1 (Fu et al. 2012).

Functional annotations were assigned to the final set of predicted genes using eggNOG-mapper v2 (Cantalapiedra et al. 2021), which examines orthologous gene clustering through the detection of orthologous groups, and InterProScan v5.61-93 (Jones et al. 2014), which performs annotation of protein family and domain information through integration of protein signatures. Functional annotations from both sources were then integrated using annotate from funannotate v1.7.4 (https://github.com/nextgenusfs/funannotate) to produce a final annotation file that was then assessed for BUSCO completeness.

### Comparative Analysis

To further assess the final set of functionally annotated genes, the number of shared functional genes with other octocorals was compared. The proteomes of *D. gigantean* (Jeon et al. 2019) (GCF_004324835.1) and *S. pistillata* (Voolstra et al. 2017) (GCF_002571385.1) were downloaded from the NCBI database, and annotations were clustered by 90% similarity using CD-HIT (Fu et al. 2012). The number of shared homologous genes was assessed using BlastP (Altschul et al. 1990) to the *E. verrucosa* genome. Annotations of predicted protein-coding genes were identified using the eggNOG v2 and InterProScan v5.61-93. A recent genome assembly of the deep-water octocoral *Trachythela* sp. (GCA_016169945.1) is available online, but unfortunately, arrived too late for inclusion in our comparative analysis.

## Supplementary Material

evad083_Supplementary_Data

## Data Availability

Genome annotations are available on GitHub (https://github.com/klmacleod/pinkseafan-genome). The genome assembly and its underlying data are available at the European Nucleotide Archive (ENA) under the study accession PRJEB61094. RNA-seq data used for the annotation are available at the European Nucleotide Archive (ENA) under the study accession PRJEB61418.

## References

[evad083-B1] Adachi K , Miyake H, Kuramochi T, Mizusawa K, Okumura S. 2017. Genome size distribution in phylum Cnidaria. Fish Sci. 83:107–112.

[evad083-B2] Altschul SF , Gish W, Miller W, Myers EW, Lipman DJ. 1990. Basic local alignment search tool. J Mol Biol. 215:403–410. 2231712 10.1016/S0022-2836(05)80360-2

[evad083-B3] Arizmendi-Mejía R , et al 2015. Demographic responses to warming: reproductive maturity and sex influence vulnerability in an octocoral. Coral Reefs 34:1207–1216.

[evad083-B4] Baril TJ , Imrie RM, Hayward A. 2022. Earl Grey: a fully automated user-friendly transposable element annotation and analysis pipeline. bioRxiv. 2022.06.30.498289. http://biorxiv.org/content/early/2022/07/02/2022.06.30.498289.abstract.

[evad083-B5] Brůna T , Hoff KJ, Lomsadze A, Stanke M, Borodovsky M. 2021. BRAKER2: automatic eukaryotic genome annotation with GeneMark-EP+ and AUGUSTUS supported by a protein database. NAR Genomics Bioinforma. 3:1–11. 10.1093/nargab/lqaa108 PMC7787252 33575650

[evad083-B6] Cantalapiedra CP , Hern̗andez-Plaza A, Letunic I, Bork P, Huerta-Cepas J. 2021. eggNOG-mapper v2: functional annotation, orthology assignments, and domain prediction at the metagenomic scale. Mol Biol. Evol. 38:5825–5829. 34597405 10.1093/molbev/msab293 PMC8662613

[evad083-B7] Chimienti G , Biologia D, Orabona V. 2020. Vulnerable forests of the pink sea fan *Eunicella verrucosa* in the Mediterranean sea. Diversity (Basel). 12:176.

[evad083-B8] Chimienti G , Mastrototaro F, D’Onghia G. 2019. Mesophotic and deep-sea vulnerable coral habitats of the Mediterranean sea: overview and conservation perspectives. Adv Stud Benthic Zo. 20:82–92.

[evad083-B9] Coz R , et al 2012. Development of a new standardised method for sustainable monitoring of the vulnerable pink sea fan *Eunicella verrucosa*. Mar Biol. 159:1375–1388.

[evad083-B10] Crisci C , et al 2017. Regional and local environmental conditions do not shape the response to warming of a marine habitat-forming species/631/158/631/208/457/45/23/45/141 article. Sci Rep. 7:1–13. 28698582 10.1038/s41598-017-05220-4 PMC5505982

[evad083-B11a] Dainat J. AGAT: Another Gff Analysis Toolkit to handle annotations in any GTF/GFF format. Version v0.8.0. Zenodo. 10.5281/zenodo.3552717

[evad083-B11] Dobin A , et al 2013. STAR: ultrafast universal RNA-seq aligner. Bioinformatics 29:15–21. 23104886 10.1093/bioinformatics/bts635 PMC3530905

[evad083-B12] Donelson JM , et al 2019. Understanding interactions between plasticity, adaptation and range shifts in response to marine environmental change. Philos Trans R Soc B Biol Sci. 374:20180186. 10.1098/rstb.2018.0186 PMC6365866 30966966

[evad083-B13] Ferrier-Pages C , et al 2009. Physiological response of the symbiotic gorgonian *Eunicella singularis* to a long-term temperature increase. J Exp Biol. 212:3007–3015. 19717684 10.1242/jeb.031823

[evad083-B14] Flynn JM , et al 2020. Repeatmodeler2 for automated genomic discovery of transposable element families. Proc Natl Acad Sci U S A. 117:9451–9457. 32300014 10.1073/pnas.1921046117 PMC7196820

[evad083-B15] Fu L , Niu B, Zhu Z, Wu S, Li W. 2012. CD-HIT: accelerated for clustering the next-generation sequencing data. Bioinformatics 28:3150–3152. 23060610 10.1093/bioinformatics/bts565 PMC3516142

[evad083-B16] Guan D , et al 2020. Identifying and removing haplotypic duplication in primary genome assemblies. Bioinformatics 36:2896–2898. 31971576 10.1093/bioinformatics/btaa025 PMC7203741

[evad083-B17] Haguenauer A , Zuberer F, Ledoux JB, Aurelle D. 2013. Adaptive abilities of the Mediterranean red coral *Corallium rubrum* in a heterogeneous and changing environment: from population to functional genetics. J Exp Mar Bio Ecol. 449:349–357.

[evad083-B18] Hall-Spencer JM , Pike J, Munn CB. 2007. Diseases affect cold-water corals too: *Eunicella verrucosa* (Cnidaria: Gorgonacea) necrosis in SW England. Dis Aquat Organ. 76:87–97. 17760382 10.3354/dao076087

[evad083-B19a] Hayward P , Ryland J. 2017. Handbook of the marine fauna of north-west Europe. 2nd ed. Oxford: Oxford University Press.

[evad083-B19] Holland LP , Dawson DA, Horsburgh GJ, Krupa AP, Stevens JR. 2013. Isolation and characterization of fourteen microsatellite loci from the endangered octocoral *Eunicella verrucosa* (Pallas 1766). Conserv Genet Resour. 5:825–829.

[evad083-B20] Holland LP , Jenkins TL, Stevens JR. 2017. Contrasting patterns of population structure and gene flow facilitate exploration of connectivity in two widely distributed temperate octocorals. Heredity (Edinb). 119:35–48. 28295035 10.1038/hdy.2017.14 PMC5520136

[evad083-B21] Hoppit G , Schmidt DN, Brazier P, Mieszkowska N. 2022. Nature-based solutions are marine protected areas an adaptation measure against climate change impacts on coastal ecosystems ? A UK case study. Nature-Based Solut. 2:100030.

[evad083-B22] Jenkins TL , Ellis CD, Triantafyllidis A, Stevens JR. 2019. Single nucleotide polymorphisms reveal a genetic cline across the North-East Atlantic and enable powerful population assignment in the European lobster. Evol Appl. 12:1881–1899. 31700533 10.1111/eva.12849 PMC6824076

[evad083-B23] Jenkins TL , Stevens JR. 2018. Assessing connectivity between MPAs: selecting taxa and translating genetic data to inform policy. Mar Policy. 94:165–173.

[evad083-B24] Jenkins TL , Stevens JR. 2022. Predicting habitat suitability and range shifts under projected climate change for two octocorals in the north-east Atlantic. PeerJ 10:e13509. 35651748 10.7717/peerj.13509 PMC9150690

[evad083-B25] Jeon Y , et al 2019. The draft genome of an octocoral. Dendronephthya gigantea. 11:949–953. 10.1093/gbe/evz043 PMC6447388 30825304

[evad083-B26] Jones P , et al 2014. Interproscan 5: genome-scale protein function classification. Bioinformatics 30:1236–1240. 24451626 10.1093/bioinformatics/btu031 PMC3998142

[evad083-B27] Kolmogorov M , Yuan J, Lin Y, Pevzner PA. 2019. Assembly of long, error-prone reads using repeat graphs. Nat Biotechnol. 37:540–546. 30936562 10.1038/s41587-019-0072-8

[evad083-B28] Laetsch DR , Blaxter ML, Leggett RM. 2020. Blobtools : interrogation of genome assemblies [version 1 ; peer review : 2 approved with reservations]. F1000Res. 6:1287.

[evad083-B29] Ledoux JB , et al, 2020. The genome sequence of the octocoral Paramuricea clavata—a key resource to study the impact of climate change in the Mediterranean. bioRxiv. 10:2941–2952.

[evad083-B30] McFadden CS , Van Ofwegen LP, Quattrini AM. 2022. Revisionary systematics of Octocorallia (Cnidaria: Anthozoa) guided by phylogenomics. Bull Soc Syst Biol. 1:1–79.

[evad083-B31] Oualid JA , Alla AA, Moukrim A, Lopez-Gonzalez PJ. 2023. Northward range expansion of *Leptogorgia dakarensis* and *Eunicella racemosa* (Cnidaria : Gorgoniidae : Anthozoa) in the Eastern Atlantic. J Mar Biol Assoc United Kingdom. 103:1–7.

[evad083-B32] Pikesley SK , et al 2016. Pink sea fans (*Eunicella verrucosa*) as indicators of the spatial efficacy of marine protected areas in southwest UK coastal waters. Mar Policy. 64:38–45.

[evad083-B33] Quinlan AR , Hall IM. 2010. BEDTools: a flexible suite of utilities for comparing genomic features. Bioinformatics 26:841–842. 20110278 10.1093/bioinformatics/btq033 PMC2832824

[evad083-B34] Readman JAJ , Hiscock K. 2017. Eunicella verrucosa Pink sea fan. In: Tyler-Walters H, Hiscock K, editors. Marine life information network: biology and sensitivity key information reviews, [online]. Plymouth: Marine Biological Association of the United Kingdom. p. 1–21.

[evad083-B35] Sartoretto S , Francour P. 2012. Bathymetric distribution and growth rates of *Eunicella verrucosa* (Cnidaria: Gorgoniidae) populations along the Marseilles coast (France). Sci Mar. 76:349–355.

[evad083-B36] Simão FA , Waterhouse RM, Ioannidis P, Kriventseva E V, Zdobnov EM. 2015. BUSCO: assessing genome assembly and annotation completeness with single-copy orthologs. Bioinformatics 31:3210–3212. 26059717 10.1093/bioinformatics/btv351

[evad083-B37] Stanke M , Schöffmann O, Morgenstern B, Waack S. 2006. Gene prediction in eukaryotes with a generalized hidden Markov model that uses hints from external sources. BMC Bioinformatics 7:62. 16469098 10.1186/1471-2105-7-62 PMC1409804

[evad083-B38] Storer J , Hubley R, Rosen J, Wheeler TJ, Smit AF. 2021. The Dfam community resource of transposable element families, sequence models, and genome annotations. Mob DNA. 12:2. 33436076 10.1186/s13100-020-00230-y PMC7805219

[evad083-B39] Tarailo-Graovac M , Chen N. 2009. Using RepeatMasker to identify repetitive elements in genomic sequences. Curr Protoc Bioinformatics. Chapter 4:4.10.1–4.10.14. 10.1002/0471250953.bi0410s25 19274634

[evad083-B40] Voolstra CR , et al 2017. Comparative analysis of the genomes of *Stylophora pistillata* and *Acropora digitifera* provides evidence for extensive differences between species of corals. Sci Rep. 7:17583. 29242500 10.1038/s41598-017-17484-x PMC5730576

[evad083-B41a] Wood C . 2013. Seasearch guide to sea anemones and corals of Britain and Ireland. Plymouth: Wild Nature Press.

[evad083-B41] Zhou Y , et al 2021. The first draft genome of a cold-water coral *Trachythela* sp. (Alcyonacea: Stolonifera: Clavulariidae). Genome Biol Evol. 13:evaa265. 10.1093/gbe/evaa265 PMC7875002 33331875

