# Supplementary material for: The First Genome of the Cold-Water Octocoral, the Pink Sea Fan, Eunicella verrucosa
Source: Genome Biol Evol. 2023 May 20;15(6):evad083. doi: 10.1093/gbe/evad083 (PMC10243833; doi:10.1093/gbe/evad083)
Supplement: evad083_Supplementary_Data [file evad083_supplementary_data.docx]

# Supplementary material

## Materials and Methods

Six pink sea fan colonies were collected via SCUBA at 8-12 metres depth from Plymouth Sound, England (lat. 50.33, long. -4.14) under MMO licence (L/2019/00143). Colonies were sampled using sea snips then immediately transferred into individual sea water-filled plastic sampling bags once at the surface. These were then immediately transported on ice to the Aquatic Resources Centre (ARC) at the University of Exeter and placed in a 350L artificial seawater tank. To enable colonies to stabilise after transport, colonies were tied to a monofilament line and held upside down within the holding tank for 24 hours. Colonies were then attached to ceramic tiles using epoxy resin putty and left to acclimatise to *ex situ* conditions at 14.3 ^o^C (+/- 0.5 ^o^C) for 16 months before being fragmented into individually 10 cm colonies, hereafter referred to as fragments, each attached to a coral plug using epoxy putty resin and colour coded to mother colony ID.

All colonies were kept unfed for seven days prior to DNA extraction. An apical 2 cm tissue clipping (with ~15-20 polyps) was taken from a live pink sea fan colony and rinsed in 70% ethanol. The polyps were scraped off with a sterile scalpel and placed into EDTA prior to extraction. Genomic DNA was extracted using the following salting-out protocol adopted from Jenkins et al. 2019:

**Salting-out protocol for Extracting Genomic DNA from Lobster Pleopods/Pink sea fan polyps**

**Materials**

Proteinase K (20 mg/ml)

RNase A (100 mg/ml)

1 % SDS cell lysis buffer (100mM Tris-Cl; 50 mM EDTA; 1 % SDS)

7.5 M ammonium acetate

0.5 M EDTA

Nuclease-free water

**Equipment**

TissueLyser & microbeads

1.5 ml microcentrifuge tubes

Sterile blue roll

Microcentrifuge & vortexer

**Protocol**

1. Remove sample from preservative and dap on sterile blue roll to remove excess ethanol.
2. Add samples (up to 30 mg) to 1.5 ml microcentrifuge tubes containing a microbead.
3. Homogenise samples by placing in a TissueLyser for 30 seconds at 30 Hz/s (repeat if necessary).

**Digestion**

1. Add the following to each tube:
   1. 350 µl 1 % SDS cell lysis buffer
   2. 42 µl 0.5 M EDTA
   3. 10 µl proteinase K.
2. Mix by vortexing and incubate at 65^o^C for 2.5 to 3 hours.
3. Add 2 µl RNase A and incubate on a thermomixer at 37^o^C for 30 minutes.

**Remove proteins and cellular debris**

1. Add 140 µl 7.5 M ammonium acetate to each tube. Mix by vortexing. Incubate at 4^o^C for 10 minutes.
2. Centrifuge at 12,000 g for 10 minutes.
3. Transfer supernatant to a new 1.5 ml microcentrifuge. Discard the previous tube.
4. Repeat steps 7-9.

**Precipitation of DNA**

1. Add 680 µl cold isopropanol (volume ratio 1:1). Mix by inverting gently 50 times. Centrifuge at 8000 g for 5 minutes.
2. Carefully discard the supernatant, avoiding contact with the pellet. Drain the tube by placing on sterile blue roll, taking care that the pellet remains in the tube.

**Washing of DNA**

1. Add 400 µl 70 % ethanol. Invert the tube several times to wash the DNA pellet. Centrifuge at 8000 g for 1 minute.
2. Carefully discard the supernatant, avoiding contact with the pellet. If a lot of supernatant remains, pulse centrifuge the tubes and discard the supernatant using a smaller pipette, again avoiding contact with the pellet.
3. Allow to air dry to 10-20 minutes. Non-contaminated pellets will turn more transparent as they dry. Avoid over-drying the DNA pellet, as the DNA will be difficult to dissolve.

**Rehydration of DNA**

1. Re-suspend dried pellets with 100 µl nuclease-free water. Invert tube to mix and spin down using centrifuge.
2. Incubate at room temperature for 30 minutes or incubate in the fridge overnight.
3. Briefly pulse centrifuge tubes and store at -20 ^o^C.

To assist with the annotation of the genome, RNAseq data was collected via an *ex situ* thermal exposure experiment.

Briefly, fragments were placed into 1062 ml individual tanks with an airline for aeration and to create water circulation. Tanks were placed in three separate water baths (hereafter referred to as bench) set to control temperature and left to acclimatise for one day. Following acclimation, TECO chillers were used to control bench water temperature; for the ‘high’ exposure condition, water was raised to 19.5 ^o^C (+/- 1 ^o^C), whilst for the ‘low’ exposure condition bench water temperature was decreased to 7 ^o^C (+/- 1 ^o^C), the control bench remained at control temperature. Once at temperature, fragments were kept at experimental condition for 24 hours, temperature was logged every 15 minutes using Tiny Tag Aquatic 2 TG-4100 data loggers. Following 24 hours, fragments were collected into 15 ml tubes and immediately placed into liquid nitrogen, before long-term storage at -80 ^o^C.

RNA was extracted via QIAzol and 1-Bromo-3-chloropropane (BCP) for phase separation followed by purification with 75% ethanol and RNA elution in nuclease-free H20 - see full protocol below.

**Protocol for RNA extraction from pink sea fan, *Eunicella verrucosa*.**

**Equipment and Reagents**

QIAzol Reagent

1-Bromo-3-chloropropane (BCP)

Isopropanol

75% ethanol in DEPC-treated water

Liquid nitrogen

Stainless steel beads

Sterile-RNase-free pipettes and tips

10 % bleach

70% ethanol

Tissue homogeniser

Vortex

Temperature-controlled Centrifuge

Hotplate

Wide-based 1.5 ml Eppendorf tubes

**Protocol**

1. Set centrifuge to 4 ^o^C

2. Clean all pipettes, workbench, scales and fume hood with 10% bleach and then 70% ethanol

3. Prepare falcon tubes containing 80 ml of 10% bleach, 70% ethanol and 2 x DEPC-treated water

4. Clean forceps and scissors in each falcon tube with reagents prepared above, in that order

5. Prepare desired number of wide-based 1.5 ml Eppendorf tubes on clean work bench, label and place onto ice

**Tissue homogenisation**

6. Remove pink sea fan-containing tube from -80 ^o^C storage and place into liquid nitrogen-filled Dewar

7. Remove pink sea fan tube from the liquid nitrogen with long forceps and place onto ice

8. Cut 50-100 mg of frozen tissue and place into prepared Eppendorf on ice.

9. Repeat for all samples to be extracted

10. Add 1 ml (1000 µl) of QIAzol to each RNA extraction prepared Eppendorf

11. Using sterile, small forceps add one stainless steel bead to each Eppendorf tube

12. Homogenise samples for 40 secs at 30 h^-s^ – check to see if all tissue is homogenised, may need longer or better positioning of the stainless steel bead.

13. Incubate samples at room temperature for 5 minutes

**Phase separation**

14. Add 100 µl BCP to each reaction, vortex gently and leave to stand at room temperature for 10 minutes

15. Centrifuge for 13 mins at 12 RPM at 4 ^o^C

16. Whilst spinning, make new Eppendorf tubes and fill with 500 µl cold isopropanol, place on ice

**RNA precipitation**

17. Transfer aqueous phase to 500 ml cold isopropanol tube and vortex for 5 secs then incubate at room temperature for 7 mins

18. Centrifuge for 10 mins at 12 RPM at 4 ^o^C

**Washing of RNA**

19. Remove isopropanol and wash with 1000 µl 75% ethanol. It is important to dislodge the pellet by flicking the tube

20. Centrifuge for 5 mins at 12 RPM

21. Repeat steps 19 and 20 to perform another wash

22. Remove all ethanol, briefly spin to ensure all ethanol is removed, leave eppendorfs open to allow last ethanol to evaporate

23. Once pellet is starting to dry (should start turning translucent) elute in 50 µl of nuclease-free H_2_0 and incubate at 50 ^o^C for 5 mins, ideally on a heated rocker plate

24. Store at -80 ^o^C

**Important that the chemical ratio is 1 QIAzol:0.1 BCP:0.5 isopropanol**

Homogenisation should be enough to break up the pink sea fan fragment and open the cells but not to completely homogenise the tissue to a fine grain as this will be of detriment to the RNA.

Extractions were quantified by Qubit RNA BR (Thermo Fisher Scientific) and quality was assessed via the Agilent 4200 Tapestation system with RNA Integrity Number (RIN); only samples with a RIN>8 were used. RNA-seq libraries were prepared from total RNA using the NEB Next® Ultra™ RNA Library Prep Kit. For RNA library preparation, Messenger RNA was purified from total RNA using poly-T oligo-attached magnetic beads; the first strand cDNA was synthesized using random hexamer primers followed by the second strand cDNA synthesis. The library was ready after end repair, A-tailing, adapter ligation, size selection, amplification, and purification. Reads were cleaned by removing base calls with a Phred score <30 using the programme fastp (Chen et al. 2018) and performing PolyG tail and PolyX tail trimming. RNA quantity was checked with a Qubit fluorometer, real-time PCR was used for quantification and an Agilent 5400 Fragment Analyzer for size distribution detection.

# Results

Manual cut-offs were selected using the histogram of read depth produced from the *hist_ploy.py* analysis using programme purge_dups v1.2.6 (Guan et al. 2020).


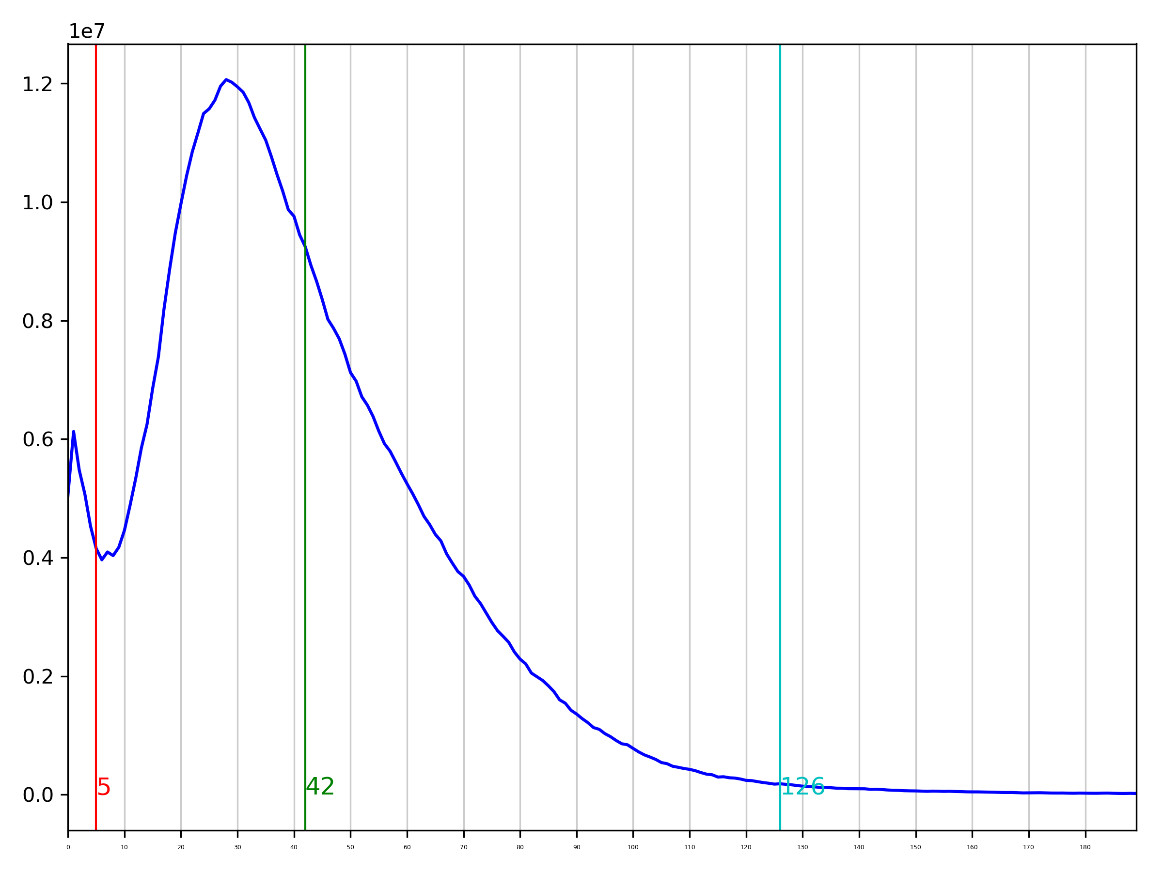


**Figure S1** Manual cut-offs were selected using the histogram of read depth produced from the *hist_ploy.py* analysis. Manual cut-offs chosen were: low = 5, midpoint = 28, high = 126. Analysis performed using purge_dups v1.2.6.


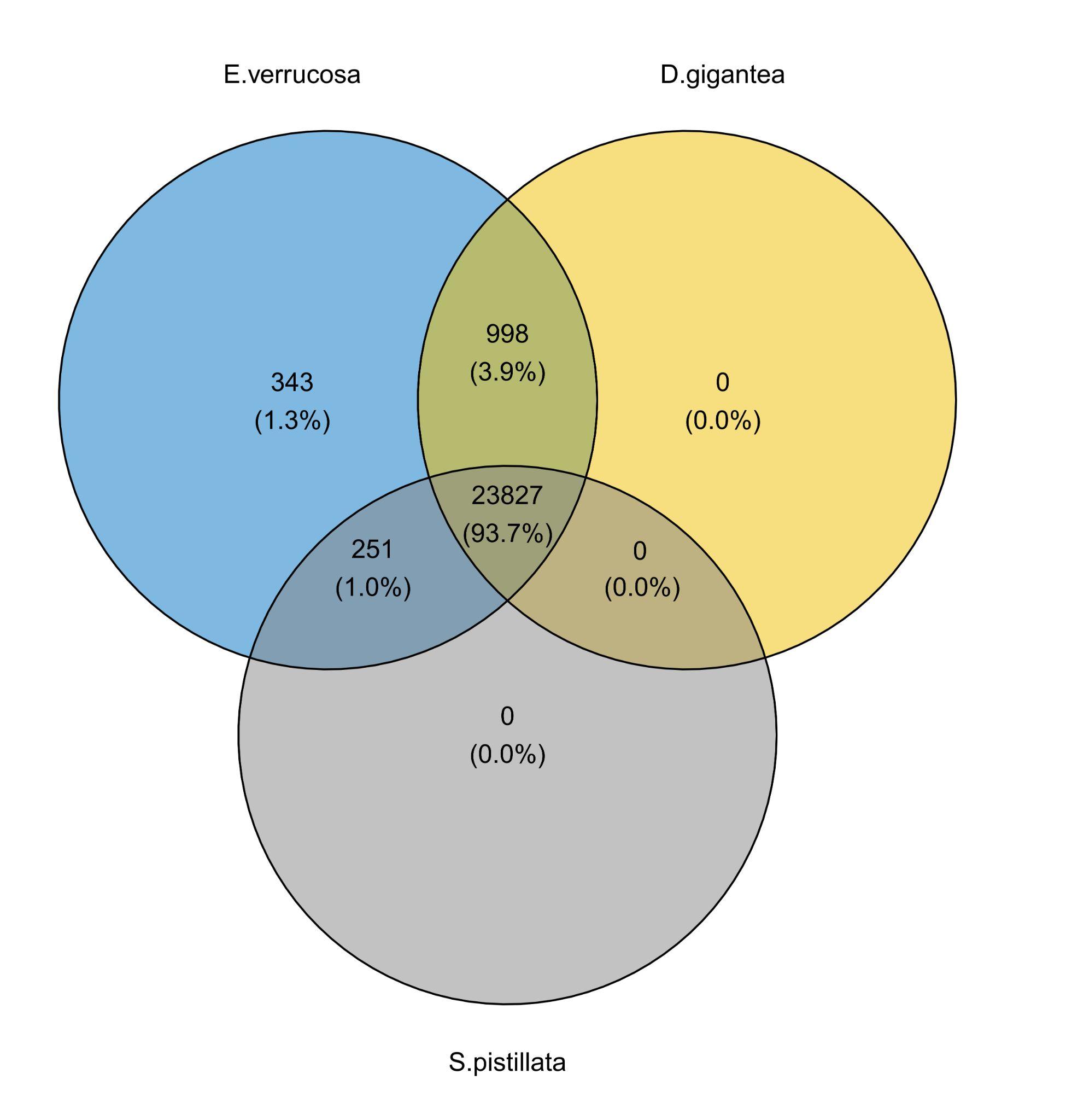


**Figure S2** Comparison of the number of shared functionally annotated genes following a blastp of *E. verrucosa* 90% similarity gene set against the 90% similarity-filtered proteomes of octocoral species *D. gigantean* and a more distantly related stony coral *S. pistillata.* Functional annotations were assigned using eggNOG-mapper v2 (Cantalapiedra et al. 2021) and InterProScanand v5.61-93 (Jones et al. 2014).


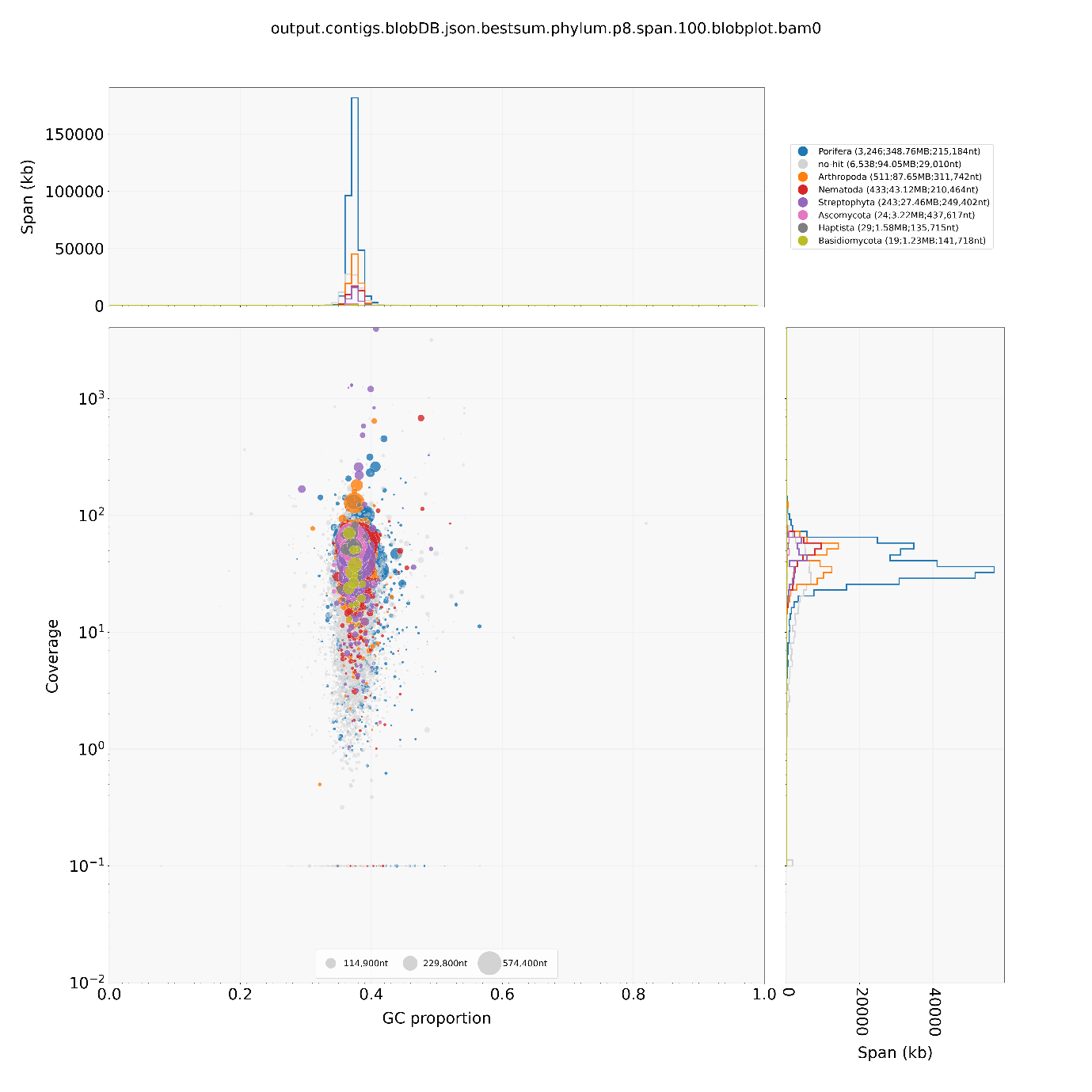


**Figure S3** Non-purged raw assembly contamination assessment with BlobTools v1.0.1 (Laetsch et al. 2020).

**
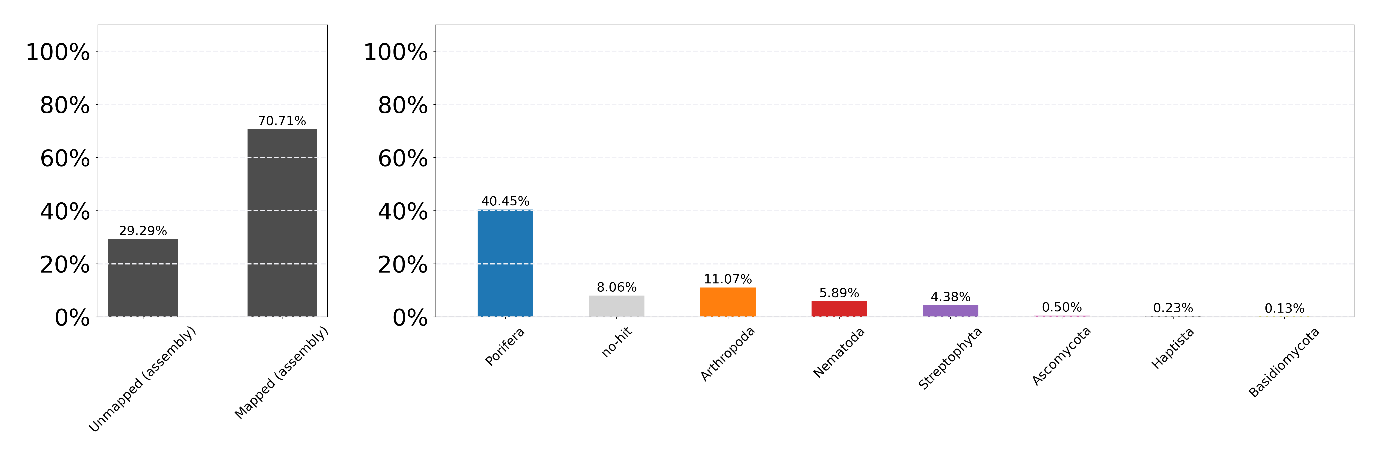
**

**Figure S4** Non-purged raw assembly contamination categorisation with BlobTools.

**Table S1** Gene set annotation statistics

| Gene set | Annotated genes | BUSCO genes (%) | | |
| --- | --- | --- | --- | --- |
|  |  | **Complete** | **Single-copy** | **Duplicated** |
| Raw annotation | 41,912 | 92.2 | 85.1 | 7.1 |
| Longest isoform | 40,003 | 92.3 | 88.4 | 3.9 |
| 90% similarity | 36,099 | 92.2 | 89.9 | 2.3 |
| Functional | 25,419 | 92.3 | 88.6 | 3.7 |
|  |  |  |  |  |

**Table S2** Summary statistics of repetitive sequences from Earl Grey (Baril et al. 2022)

| **Category** | **Total repeat length (bp)** | **% of assembly** |
| --- | --- | --- |
| DNA | 54,233,114 | 11.59 |
| LINE | 26,477,500 | 5.66 |
| SINE | 2,837,057 | 0.61 |
| LTR | 30,757,273 | 6.58 |
| Penelope | 9,918,943 | 2.12 |
| Rolling circle | 3,906,347 | 0.84 |
| Unclassified | 85,390,169 | 18.3 |
| Non-repeat | 252,794,816 | 54.04 |

# References

Baril TJ, Imrie RM, Hayward A. 2022. Earl Grey: a fully automated user-friendly transposable element annotation and analysis pipeline. *bioRxiv*. 2022.06.30.498289. http://biorxiv.org/content/early/2022/07/02/2022.06.30.498289.abstract.

Cantalapiedra CP, Hern̗andez-Plaza A, Letunic I, Bork P, Huerta-Cepas J. 2021. eggNOG-mapper v2: Functional Annotation, Orthology Assignments, and Domain Prediction at the Metagenomic Scale. *Mol. Biol. Evol.* 38:5825–5829. doi: 10.1093/molbev/msab293.

Chen S, Zhou Y, Chen Y, Gu J. 2018. Fastp: An ultra-fast all-in-one FASTQ preprocessor. *Bioinformatics*. 34:i884–i890. doi: 10.1093/bioinformatics/bty560.

Guan D et al. 2020. Identifying and removing haplotypic duplication in primary genome assemblies. *Bioinformatics*. 36:2896–2898. doi: 10.1093/bioinformatics/btaa025.

Laetsch DR, Blaxter ML, Leggett RM. 2020. BlobTools : Interrogation of genome assemblies [ version 1 ; peer review : 2 approved with reservations ]. 1287:1–16.

Jenkins TL, Ellis CD, Triantafyllidis A, Stevens JR. 2019. Single nucleotide polymorphisms reveal a genetic cline across the north-east Atlantic and enable powerful population assignment in the European lobster. *Evol. Appl.* 12:1881–1899. doi: 10.1111/eva.12849.

Jones P, Binns D, Chang HY, Fraser M, Li W, McAnulla C, McWilliam H, Maslen J, Mitchell A, Nuka G, Pesseat S, Quinn A, Sangrador-Vegas A, Scheremetjew M, Yong SYm Lopez R, Hunter S. 2014. InterProScan 5: Genome-scale protein function classification. *Bioinformatics.* 30:1236-1240. [doi.org/10.1093/bioinformatics/btu031](https://www.zotero.org/google-docs/?mykDzW)
